# The Effectiveness of Audio Recordings in Aiding Students' Recall of Biochemistry Concepts: A Quasi-Experimental Study

**DOI:** 10.7759/cureus.87876

**Published:** 2025-07-14

**Authors:** Jamuna Rani Ayyalu, Venkata BharatKumar Pinnelli, Surendra Babu T, Aga Ammar Murthuza, Venkataramana Kandi

**Affiliations:** 1 Biochemistry, Sree Balaji Medical College and Hospital, Chennai, IND; 2 Biochemistry, Vydehi Institute of Medical Sciences and Research Centre, Bangalore, IND; 3 Anatomy, Vydehi Institute of Medical Sciences and Research Centre, Bangalore, IND; 4 Clinical Microbiology, Prathima Institute of Medical Sciences, Karimnagar, IND

**Keywords:** audio recordings, bachelor of medicine and bachelor of surgery (mbbs), biochemistry, multiple-choice questions (mcqs), understanding

## Abstract

Introduction

Engaging students in the study of biochemistry requires the use of innovative teaching and learning (TL) strategies. It is essential to capitalize on the current generation's growing use of microphones by offering audio recordings that address key biochemistry concepts. Allowing students to listen to the audio recordings during study sessions could enhance comprehension and memory. This study aimed to assess the efficacy of audio recordings as an additional TL tool to help students remember biochemistry concepts.

Methods

Two hundred and fifty first-year Bachelor of Medicine and Bachelor of Surgery (MBBS) students from the 2022-2023 cohort at a tertiary care teaching institution in Chennai, India, received an audio recording summarizing important aspects after a topic was covered in the classroom. A Google Form with five multiple-choice questions (MCQs) via Google Classroom (California, United States) and a shared WhatsApp group (California, United States) was used to evaluate the usefulness of these audio resources. Students were asked to rate how helpful they thought the audio recordings were for remembering biochemistry concepts on a five-point Likert scale.

Results

After listening to the audio recordings, more than 92% (230/250) of students answered the MCQs correctly, compared to 54% (135/250) of students before listening to the audio recordings. After listening to the audio recordings, the mean score on the subsequent MCQ test increased considerably to 17.64±2.02 (p<0.0001) from 8.16±3.08 before the intervention.

Conclusion

The results indicate that audio recordings are a useful and affordable way to improve understanding of biochemistry concepts. Student acceptance of this alternate TL approach is high, and it holds promise for future advancements in teaching methods.

## Introduction

Technological developments and the growing need for creative teaching and learning (TL) approaches have caused major changes in the educational landscape in recent years. Using audio-based TL resources, such as audiobooks and audio recordings, has become one of these technologies' most promising practices to improve student engagement and comprehension, especially in challenging disciplines like biochemistry. Given that the present generation of students is more used to obtaining information via digital media, such as podcasts and audiobooks, this change is especially pertinent [1].

According to research, using audio resources to accommodate a variety of learning preferences and styles can help students learn more effectively. Auditory learning, for example, can greatly enhance comprehension and recall of difficult concepts. Since students can reinforce their information through many channels, it has been discovered that using both visual and audio modalities simultaneously improves comprehension [2]. Cognitive load theory holds that learning is more successful when information is provided to minimize unnecessary cognitive burden, and this multimodal technique is consistent with its tenets [3]. Medical education benefits greatly from auditory pathways since they help students retain information, especially those who learn best by hearing. Medical students can improve their learning and memory by using strategies like audio material [4].

The difficulties in explaining complex ideas and metabolic processes in the context of biochemistry instruction can frequently cause students to become disinterested and frustrated. Conventional instructional techniques, such as lectures and textbook readings, might not fully meet the various demands of students [5]. In response, teachers have started looking into different teaching methods, such as audio snippets highlighting important ideas and encouraging active learning [6]. Teachers can create a more adaptable and stimulating learning environment by giving students audio resources they can listen to at their convenience.

Studies have shown that audio-based learning resources are beneficial. Evenddy et al., for instance, emphasized the advantages of project-based learning in higher education and the significance of involving students creatively [7]. Alshehhi et al., in their research, also showed that game-based learning techniques greatly increase student enthusiasm and engagement, indicating that interactive and multimedia methods can result in better learning outcomes [8].

Additionally, incorporating audio resources is consistent with constructivist learning ideas, which promote student cooperation and active engagement [9]. Teachers can produce a more engaging and dynamic educational environment by enabling students to listen to audio material during group assignments or conversations. In addition to encouraging deeper comprehension, this method empowers students to take charge of their education [5].

Audio-based TL resources can satisfy the need for customized TL experiences. Students' perceptions of generative artificial intelligence (AI) in the classroom indicate they greatly favor personalized learning support [10]. Teachers can provide personalized support and accommodate various TL styles, like using audio resources, which increases student motivation, engagement, and concept retention. Through this study, we aimed to assess the efficacy of audio recordings as an additional TL tool to help students remember biochemistry concepts.

## Materials and methods

This study employed a quasi-experimental design to evaluate the effects of audio recordings and resources as an extra TL tool that aids students' retention of biochemistry concepts. The study included 250 Bachelor of Medicine and Bachelor of Surgery (MBBS) students from the 2022-2023 batch. A homogeneous group with comparable academic levels was ensured while the study participants were selected. All participants gave their informed consent, attesting to their knowledge of the study's objectives and their freedom to discontinue participation at any moment without facing consequences. The responses of the participants were kept private during the whole investigation. The Institutional Human Ethics Committee of Sree Balaji Medical College & Hospital, Chennai, Tamil Nadu (Ref. No. 002/SBMCH/IHEC/2022/1934) approved the study.

Audio material development

Essential concepts covered in the biochemistry curriculum were summarized in seven-to-eight-minute audio recordings. Because the audio information for these recordings was taken from lectures in the classroom, it closely matched the topics being learned. Clarity and comprehension of the audio recordings were ensured by recording them in a special soundproof room [11].

Delivery method

Students accessed the audio recordings via a shared Google Drive (Google LLC) linked to a shared Gmail (Google LLC) account. This approach made it simple for everyone to participate, and the student attendance was recorded. The links to the audio recordings were circulated via Google Classroom (Google LLC) and a common WhatsApp (WhatsApp LLC) group, ensuring that all students could easily download the content.

Assessment of effectiveness

**Figure 1:**
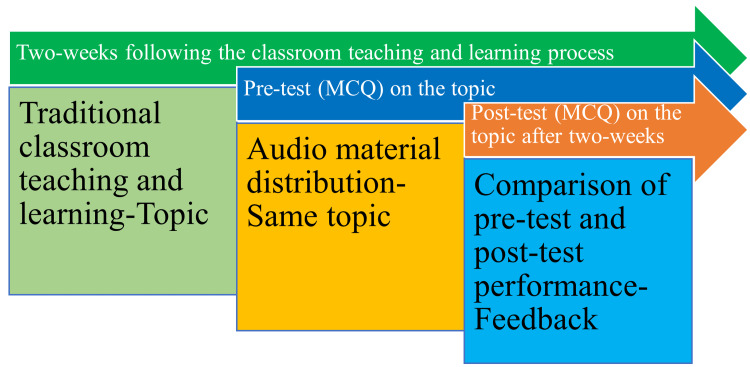
Diagrammatic representation of the study methodology Image credit: Venkataramana Kandi. MCQ: multiple choice questions

A Google Form (Google LLC) with five multiple-choice questions (MCQs) was constructed to assess how the audio recordings improved recollection of the concepts [22]. The authors verified that the MCQs are challenging and impartial for students while also being clear, straightforward, and in line with learning goals. In order to do this, the body of question-and-answer choices was carefully crafted, taking into account the questions' difficulty and applicability. MCQs were also examined for grammar mistakes, choices that overlapped, and a fair distribution of right answers across the alternatives. The students were asked to complete the MCQs two weeks before and two weeks after listening to the audio recordings, and the scores were compared [12]. The methodology of the study has been detailed in Figure 1.

Data collection

Data were collected through the responses to the MCQs, which were designed to measure the students' recall of the biochemistry concepts [13]. The responses were analyzed quantitatively to determine the percentage of correct answers before and after the introduction of the audio recordings. Additionally, quantitative feedback was gathered from students regarding their experiences with the audio recordings, focusing on their perceived usefulness and engagement [23]. Furthermore, students were encouraged to share their experiences about the intervention through WhatsApp (WhatsApp LLC) or any convenient mode, which were recorded as qualitative feedback.

Statistical analysis

The data collected from the MCQs was analyzed using descriptive statistics to summarize the students' performance. A probability (p) value was computed to determine the degree to which the audio recordings enhanced the students' memory recall, and the results were interpreted as percentages, mean scores, and standard deviations. A p-value <0.05 was considered statistically significant. Microsoft Office 2019 Excel (Microsoft Corp., Redmond, WA, USA) was used to record the data, and IBM Corp. Released 2011. IBM SPSS Statistics for Windows, Version 20.0. Armonk, NY: IBM Corp. was used to conduct statistical analysis.

## Results

Over 92% (230/250) of students who listened to the audio material provided correct answers in the MCQs, compared to 54% (135/250) of students before the introduction of the audio recordings. The mean score on the subsequent MCQ evaluation increased considerably from 8.16±3.08 before the introduction of the audio recordings to 17.64±2.02 (p<0.0001). Several important findings were uncovered via qualitative comments in addition to the quantitative data. Regarding accessibility and engagement, 99.2% (248/250) of students said the audio segments helped them learn, comparing the experience to listening to a podcast. The flexibility of listening to the content while on the go was valued by many students, as it made their study habits easier. Students said listening to the audio recordings helped them remember difficult terms and concepts, especially those about metabolic pathways. This supports that learning through audio recordings can improve memory recall. Students strongly preferred audio learning over conventional textbook techniques in their input on the subject. According to the students, the audio material made learning more interesting and was a pleasant diversion from thick textbooks. Despite the generally favorable feedback, some students recommended adding more interactive components to the audio recordings, like tests or discussion starters, to improve comprehension and engagement even more (Table 1).

**Table 1:** Student feedback on the utility of audio material in learning TL: teaching and learning

Study participant	Feedback on the intervention
Student 1	Simple approach to listen, learn, and remember concepts. It is just like listening to a story and is more convenient than reading books. Such TL materials should be provided for all important topics/subjects
Student 2	I used the recording and made notes out of it, and I plan to use it during the exams
Student 3	It is similar to listening to a podcast, in which we understand all that is spoken rather than merely reading without comprehending
Student 4	I believe we need this kind of strategy in the long run. It has also been demonstrated in the past wherein the ancient Gurukul system lacked written texts. The knowledge transfer was entirely auditory
Student 5	I think the concept of recording audio was incredibly helpful and lessened the learning load for some important and challenging subjects

## Discussion

In recent years, there has been an increase in interest in audio-based resources, such as audiobooks and audio recordings, in TL methods, especially with challenging disciplines like biochemistry. This study sought to determine how well audio clips improved students' understanding and memory of biochemistry concepts. The results of this study are comparable to earlier research showing how audio-visual aids improve student learning outcomes. Pascasie et al., for example, discovered that audio-visual aids greatly improve academic performance in geography classes, indicating that such advantages may be seen in other fields, such as biochemistry [14]. By appealing to wider senses, multimodal learning, made possible using audio recordings, has been demonstrated to enhance comprehension and retention [15]. This is consistent with the cognitive paradigm of multimedia learning, which holds that information presented in both visual and audio modalities helps learners comprehend it more effectively.

Besides, the Sutopo et al. study showed that audio-visual materials can raise student enthusiasm and engagement in physical education, bolstering the idea that audio-based resources might improve learning in wider dimensions [16]. The favorable comments from the study's participants, who compared the audio recordings to podcasts and valued their practicality, are consistent with research by Takaeb et al. that found that audio-visual materials greatly enhanced students' capacity for higher-order thinking [17].

One of the main benefits of audio snippets is their capacity to support repeated learning, which is essential for understanding challenging biochemical concepts. It was established that content retention and recall are improved by repeated exposure [7]. In this study, students stated that hearing the audio recordings several times improved their memory of difficult terms and ideas, especially those on metabolic pathways. Results from the Xu et al. study corroborate this finding by stressing the need for repetition in learning processes, especially in disciplines that call for in-depth comprehension [15].

The usefulness of audio recordings was also aided by their accessibility via apps like Google Drive and WhatsApp. Modern learners, who frequently look for flexible learning options, will find the ease of obtaining study materials while on the go appealing [10,18]. This accessibility is helpful for students who might find it difficult to learn using traditional techniques because it promotes individual study and lets them interact with the content at their own speed.

Including audio resources also takes into account the variety of learning styles that exist in a classroom. As Zuo et al. pointed out, diverse learning modalities can accommodate different student preferences, improving comprehension and engagement overall [18]. According to this study, pupils who listened to audio recordings showed better recall than those who followed standard textbooks. This lends credence to the idea that adding audio-visual components might make a classroom more welcoming and accommodating of various learning styles [19].

Furthermore, students' positive reactions to the audio recordings suggest they made learning simpler and more enjoyable. This agrees with the findings of Dhamayanti et al., who discovered that audio-visual materials significantly increased student motivation and engagement across different learning contexts [20]. Listening to audio recordings can help foster a more positive attitude toward learning for long-term academic success.

The study's findings have significant ramifications for curriculum designers and teachers. Given the beneficial effects of audio recordings on student learning outcomes, adding audio-based materials to the biochemistry curriculum may improve student understanding and engagement. Future studies should examine how audio-based learning resources affect students' academic performance in varied areas and educational levels. Studies could also explore how long and often audio samples should be played to get the most impact.

The results also emphasize how important it is for teachers to consider the various learning styles of their pupils when creating lesson plans. Teachers can establish a more engaging and inclusive learning environment that meets the requirements of every student by utilizing a range of audio-visual materials [21]. It has been shown that using audio snippets as an additional teaching aid in biochemistry classes can significantly improve students' retention and understanding. The huge gain in performance and the encouraging comments from students highlight how crucial it is to implement cutting-edge teaching strategies that apply available and affordable technologies to create engaging learning opportunities. Utilizing audio-visual aids will become increasingly important as teaching methods change to accommodate students' varied demands and enhance learning outcomes.

Study limitations

This research was carried out as a quick, one-time intervention. It simply included a few basic biochemistry concepts. Without a control group, the study assessed the effectiveness of the intervention using the traditional method. The study didn't investigate how the intervention affected students' theoretical and practical skills or how well they retained the concepts over time.

## Conclusions

This study highlights the significant potential of audio recordings as an innovative auxiliary tool in biochemistry training by demonstrating their value in enhancing student recall and understanding. The positive feedback from students and the significant improvements in performance metrics demonstrate the advantages of incorporating audio-based TL materials into the curriculum. As educational methods evolve, implementing such multimedia tactics can contribute to developing a more dynamic and inclusive learning environment that can suit diverse learning preferences. Future research should continue to look at the long-term benefits of audio learning tools and their applicability in different educational contexts to improve student performance.
